# Sexually Dimorphic, Developmental, and Chronobiological Behavioral Profiles of a Mouse Mania Model

**DOI:** 10.1371/journal.pone.0072125

**Published:** 2013-08-13

**Authors:** Michael C. Saul, Sharon A. Stevenson, Stephen C. Gammie

**Affiliations:** 1 Department of Zoology, University of Wisconsin–Madison, Madison, Wisconsin, United States of America; 2 Neuroscience Training Program, University of Wisconsin–Madison, Madison, Wisconsin, United States of America; Kent State University, United States of America

## Abstract

Bipolar disorders are heritable psychiatric conditions often abstracted by separate animal models for mania and depression. The principal mania models involve transgenic manipulations or treatment with stimulants. An additional approach involves analysis of naturally occurring mania models including an inbred strain our lab has recently characterized, the Madison (MSN) mouse strain. These mice show a suite of behavioral and neural genetic alterations analogous to manic aspects of bipolar disorders. In the current study, we extended the MSN strain's behavioral phenotype in new directions by examining in-cage locomotor activity. We found that MSN activity presentation is sexually dimorphic, with MSN females showing higher in-cage activity than MSN males. When investigating development, we found that MSN mice display stable locomotor hyperactivity already observable when first assayed at 28 days postnatal. Using continuous monitoring and analysis for 1 month, we did not find evidence of spontaneous bipolarism in MSN mice. However, we did find that the MSN strain displayed an altered diurnal activity profile, getting up earlier and going to sleep earlier than control mice. Long photoperiods were associated with increased in-cage activity in MSN, but not in the control strain. The results of these experiments reinforce the face validity of the MSN strain as a complex mania model, adding sexual dimorphism, an altered diurnal activity profile, and seasonality to the suite of interesting dispositional phenomena related to mania seen in MSN mice.

## Introduction

Bipolar disorders (BPDs) are heritable psychiatric disorders characterized by episodes of mania and depression [1], [2]. They are common mental health problems, exhibiting an estimated prevalence between 1% and 5% [3], [4]. These behavioral pathologies cause pain and suffering to those afflicted, including the affective oscillations typifying BPDs, side effects from mood stabilizers [5], [6], disruption of daily rhythms [7], social dysfunction [8], [9], comorbid illicit drug abuse [10], [11], psychosis [12], and excess mortality [13], [14]. Economically, BPDs have been called the most expensive behavioral health diagnoses [15]. A recent estimate of the per-patient lifetime costs of BPDs in Australia was $76,821-$134,318 AUD ($78,304-$136,910 USD) [16]. The high costs of BPDs may even be increasing; the direct and indirect US economic burden of BPDs more than doubled over 18 years from $45 billion in 1991 ($69 billion in 2012 dollars) to an estimated $151 billion in 2009 ($159 billion in 2012 dollars), a growth in costs well above that expected due to epidemiological factors [17], [18]. Though prevalence, heritability, humanistic burdens, and economic costs have made BPDs the subject of intense study by human geneticists, a convincing mechanistic molecular etiology for BPDs remains elusive due to the high likelihood of a polyvalent genotype and to the many technical challenges inherent in working with humans [19]–[21].

Animal modeling has the potential to elucidate much about BPDs and their mechanistic underpinnings. Models for BPDs typically splits these disorders into the complimentary endophenotypes of mania and depression [22], [23]. Single gene transgenics [24]–[26] and treatment with stimulant drugs [27] are the most frequent approaches to modeling mania, though for disorders as phenotypically and genetically complex as BPDs, these approaches have limitations. More recently, inbred strains naturally displaying desired endophenotypes have shown utility as models for both poles. The Flinders-sensitive line of rats, an inbred rodent model of depression, has successfully aided in the elucidation of many aspects of depression [28]–[30]. Valid inbred mania models have only recently been characterized, with the Black Swiss line advanced as a potential inbred mania model [31]. Studies on the Black Swiss strain of mice have shown that while these mice are promising, their use is subject to limitations [32], [33].

Our lab has worked to characterize an inbred mouse strain as a model for mania. The Madison (MSN) mouse strain is an inbred strain derived over a period of approximately 15 years via multiple rounds of selection from the outbred hsd:ICR (ICR) strain. A full description of the MSN breeding history appears in our previous work [34]. MSN mice are highly inbred; we have estimated their inbreeding coefficient at 0.95 [35]. MSN mice show increased in-cage activity, decreased sleeping, increased sexual behavior, and increased forced swimming relative to control mouse strains. These mania-like behaviors are not associated with an increase in anxiety measures. Further, the MSN manic phenotype is moderated by lithium chloride and olanzapine (Zyprexa) treatments [35]. The MSN strain shows a suite of brain gene expression differences consistent with BPDs. These gene expression differences imply probable genomic correlates relative to the ICR strain that include genomic loci homologous to human positions implicated in BPDs, schizophrenia, and ADHD, psychiatric disorders with related molecular correlates [36]. Together, these characteristics suggest that MSN mice share many physiological characteristics with manic aspects of human BPDs [34]. We believe the MSN strain represents a naturally occurring mania model with significant face and construct validity. Further, because our work with the MSN strain utilizes the outbred ICR strain as a natural control, experimentation with MSN mice is methodologically straightforward.

Our previous work elucidated some aspects of the MSN strain's behavior and genetics, but we have yet to answer some essential questions about the phenotype displayed by these mice. Thus far, we have concentrated on MSN males; we have little information on correlates of mania in females from this strain. We do not know the timing of the phenotype's onset during development. Though we have characterized the strain as a primarily manic model, some of our evidence suggests that MSN mice display spontaneous behavioral bipolarism, an interesting finding we have yet to investigate fully. Previous work on these animals' diurnal activity pattern has been rudimentary, and we have not examined the role of seasonality in the MSN phenotype.

The current study seeks to address these limitations of our previous behavioral phenotyping of MSN mice in four experiments. The first experiment characterizes females, seeking both to replicate our previous findings from males in female mice and to describe any sexual dimorphism. The second experiment looks for the age-of-onset of the MSN phenotype from shortly after weaning until early adulthood. The third experiment, a full 28 days of continuous video data collection on the same mice, provides information on both spontaneous behavioral bipolarism and strain diurnal activity. The fourth experiment examines seasonality as a component of the MSN phenotype, measuring behavior in different photoperiods. In each of these experiments, we use spontaneous in-cage locomotor activity as the dependent behavioral measure. We have found this to be a robust, ecologically-valid measure for observing the MSN phenotype; with modest sample sizes, we have observed that MSN mice consistently show double the in-cage activity of multiple control strains [34], [35].

## Results

### Females

We first tested whether estrous state explained variance in female in-cage activity. We found no significant effect of estrous state on in-cage activity (*F*_3, 28_ = 1.67, *p* = 0.20, η_p_^2^ = 0.152) in a one-way ANOVA on transformed in-cage activity and no significant interaction effect of strain and estrous state on in-cage activity (*F*_2, 25_ = 2.37, *p* = 0.11, η_p_^2^ = 0.159) in a two-way ANOVA on transformed in-cage activity. Consequently, we excluded estrous state from subsequent ANOVA models.

**Figure 1:**
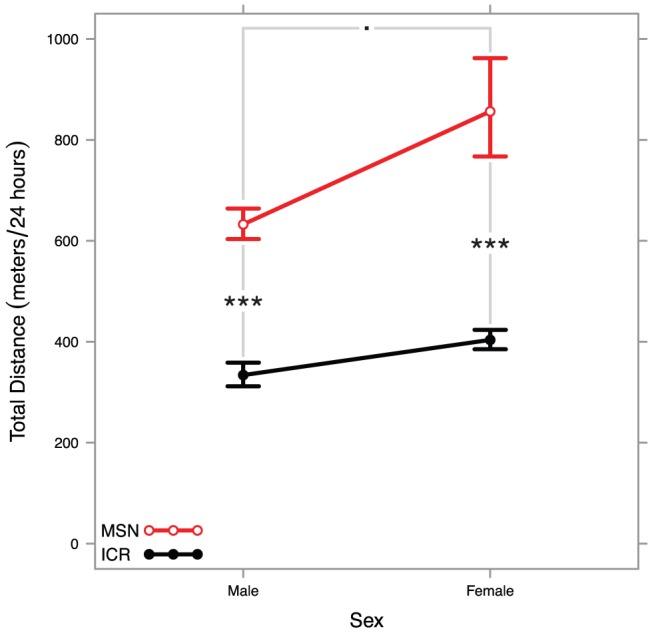
Interaction plot showing strain differences between back-transformed means and standard errors of MSN and ICR males and females. A two-way ANOVA found a highly significant strain effect, a significant sex effect, and no significant interaction effect. All significant pairwise tests are summarized in the plot (Tukey HSD: *** <*p* = 0.001≤ ** <*p* = 0.01≤ * <*p* = 0.05≤. <*p* = 0.10).

We found a highly significant strain effect (*F*_1, 60_ = 96.28, *p* = 4.4×10^−14^, η_p_^2^ = 0.616), a significant sex effect (*F*_1, 60_ = 11.44, *p* = 0.0013, η_p_^2^ = 0.160), and no significant interaction effect (*F*_1, 60_ = 0.04, *p* = 0.84, η_p_^2^ = 0.001) using a two-way ANOVA on transformed in-cage activity. The results of pairwise post-hoc tests are reported in Table S1a. All results are back-transformed and summarized in Figure 1. MSN females displayed locomotor hyperactivity relative to outbred females. Females from both strains displayed heightened in-cage activity compared to males.

A few MSN females showed 24-hour in-cage activity nearly half an order of magnitude higher than other MSN females. After inspecting the raw ethometry traces for errors, we confirmed that six MSN females were extraordinarily hyperactive, travelling over 1 km over the course of 24 hours. In all our data on males, we can confirm only a handful of isolated instances of a male animal traveling over 1 km in 24 hours and no instances of males exhibiting this extraordinary locomotor hyperactivity in the same photoperiod as the females tested here. One female travelled over 6 km in 24 hours while within a cage measuring 30.5 cm by 17.7 cm.

### Development

**Figure 2:**
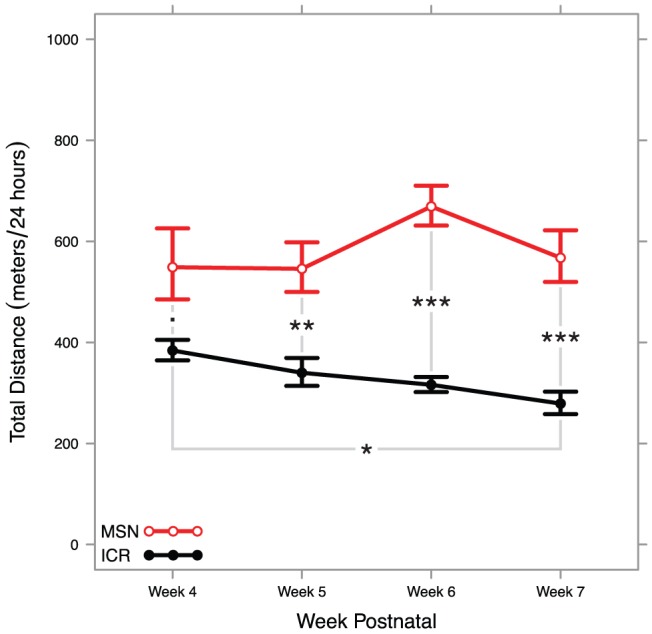
Interaction plot showing back-transformed means and standard errors of MSN and ICR mice at four different stages of early development. A two-way ANOVA found a highly significant strain effect, no significant effect of developmental stage, and a mildly significant interaction effect that may be resultant from statistical noise. All significant pairwise tests are summarized in the plot (Tukey HSD: *** <*p* = 0.001≤ ** <*p* = 0.01≤ * <*p* = 0.05≤. <*p* = 0.10).

To observe developmental time course, we recorded 24 hours of behavior each week for 8 MSN and 8 ICR mice between postnatal weeks 4 and 7. We found a highly significant strain effect (*F*_1, 56_ = 102.07, *p* = 3.2×10^−14^, η_p_^2^ = 0.646), no significant effect of week studied (*F*_3, 56_ = 1.72, *p* = 0.17, η_p_^2^ = 0.084), and a modestly significant interaction effect (*F*_3, 56_ = 2.96, *p* = 0.040, η_p_^2^ = 0.137) using a two-way ANOVA on transformed in-cage activity data. The results of pairwise post-hoc tests are reported in Table S1b. Back-transformed data are summarized in Figure 2. These results suggest that ICR in-cage activity may start higher and attenuate somewhat between weeks 4 and 7. These results also indicate that MSN mice already display hyperactivity after weaning and appear to display relatively stable locomotor hyperactivity over time.

### Month Long Observations

**Figure 3:**
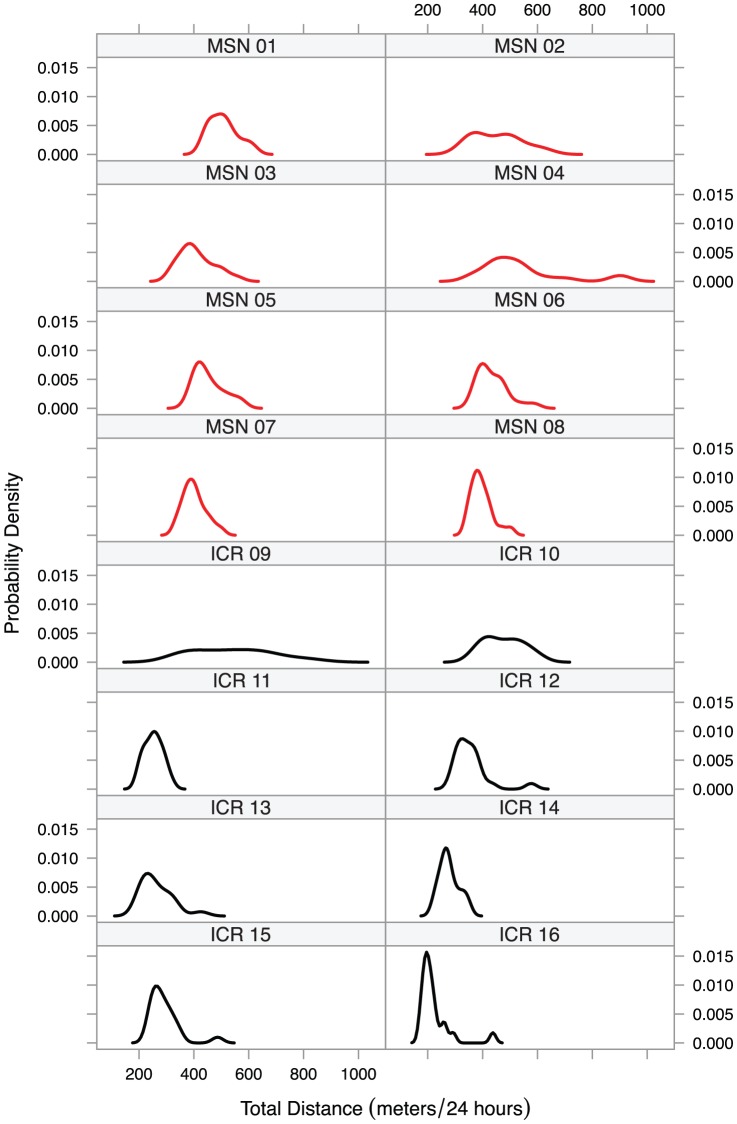
Probability density plots for distance travelled per day from each mouse in the month-long activity experiment. MSN mice showed no noticeable bipolarism relative to ICR mice.

To investigate spontaneous behavioral bipolarism in MSN mice, we observed 8 MSN and 8 ICR males continuously over 28 days between postnatal weeks 8 and 12. Figure 3 shows the probability density functions for all uninterrupted 24-hour periods for which we have data. While the MSN probability density functions are generally elevated from the ICR probability density functions, these individual probability density functions do not show the strong spontaneous bipolarism we saw in the MSN group probability density functions in our previous work [34].

**Figure 4:**
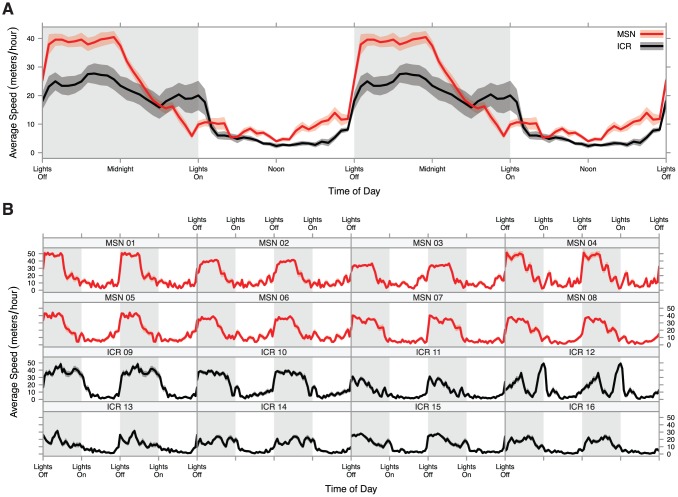
Diurnal activity plot of mice examined for a month. **A**) Half-hour in-cage activity averages for each strain with ribbons representing standard error. Diurnal activity panels are double plotted for ease of viewing. MSN mice showed a different diurnal activity pattern than ICR mice, displaying elevated activity just prior to the transition to dark period, highly elevated activity in the early dark period, and a drop in activity midway through the dark period. **B**) Half-hour in-cage activity averages for each mouse studied with ribbons representing standard error. Panels are double plotted for ease of viewing. MSN mice showed less variability in diurnal activity profile than outbred ICR mice.

We broke the month long measurements into half-hour increments for a complete and high-resolution portrait of diurnal activity profile. These observations are displayed by strain in Figure 4A, and all formal tests are available in Table S1c. We found that the MSN activity profile is very different from the ICR activity profile. MSN mice showed significantly higher in-cage activity during the second half of the light period while ICR mice were mostly still asleep (at 1500: t_10.64_ = 3.51, FDR-adjusted *p* = 0.019) and they continued to display significantly higher in-cage activity in the first half of the dark period compared to ICR mice (at 2100: t_13.73_ = 3.99, FDR-adjusted *p* = 0.013). Their in-cage activity levels fell dramatically during the second half of the dark period, showing no statistically significant difference from ICR mice starting an hour after midnight (at 0100: t_10.22_ = 1.81, FDR-adjusted *p* = 0.155), and between 4 am and lights on at 6 am, MSN mice displayed a trend toward lower in-cage activity than ICR mice (at 0530: t_7.57_ = −2.47, FDR-adjusted p = 0.077). These results clearly demonstrate that MSN mice display a response to transitions between light and dark periods, but their overall diurnal activity profile appears to display an advanced angle of photoentrainment. The MSN activity profile also appears more stereotyped than the ICR activity profile; in Figure 4B, the 8 individual MSN mice display activity profiles very similar to one another while the 8 ICR mice display idiosyncratic activity profiles that only become one general activity profile when averaged together.

### Photoperiod

**Figure 5:**
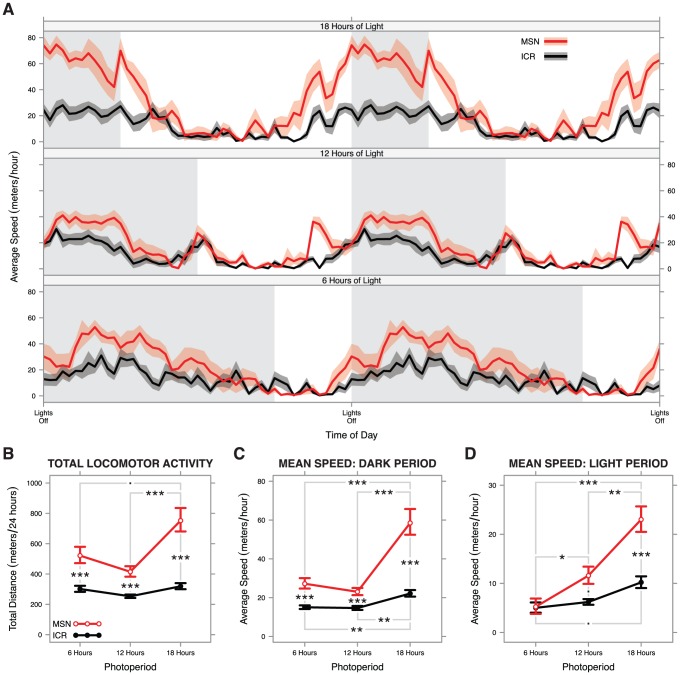
Results of photoperiod experiment. **A**) Diurnal activity plots for each photoperiod examined with lights off times aligned. Diurnal activity panels are double plotted for ease of viewing all time periods. **B**–**D**) Interaction charts showing back-transformed means and standard errors of: **B**) total 24-hour in-cage activity, **C**) mean speed during the dark period, and **D**) mean speed during the light period. Photoperiod-dependent differences in total in-cage activity were seen in MSN but not ICR mice (**B**), though photoperiod-dependent alterations to the amount of total activity budgeted in the light and dark periods were seen in both strains (**C**, **D**). All significant pairwise tests are summarized in panels **B**–**D** (Tukey HSD: *** <*p* = 0.001≤ ** <*p* = 0.01≤ * <*p* = 0.05≤. <*p* = 0.10).

After observing the refined diurnal activity profile in the month long activity study, we evaluated whether MSN mice display alterations in in-cage activity in photoperiods associated with different seasons. We raised groups of 8 MSN and 8 ICR males from weaning to 12 weeks in 3 photoperiods: 18 h (18∶6 L∶D), 12 h (12∶12 L∶D), and 6 h (6∶18 L∶D). Activity profiles when housed in these different photoperiods are displayed in Figure 5.

In the 18 h photoperiod, MSN mice exhibited very high in-cage activity during the dark period and an apparent increased in-cage activity during the light period close to the transitions (Figure 5A). This suggested a general elevation of MSN in-cage activity in long photoperiods. Formal testing of this hypothesis found a highly significant strain effect (*F*_1, 41_ = 105.72, *p* = 6.5×10^−13^, η_p_^2^ = 0.721), a highly significant photoperiod effect (*F*_2, 41_ = 13.86, *p* = 2.5×10^−5^, η_p_^2^ = 0.403), and no significant interaction effect (*F*_2, 41_ = 1.59, *p* = 0.22, η_p_^2^ = 0.001) in a two-way ANOVA on transformed 24-hour in-cage activity data. The results of pairwise post-hoc tests are reported in Table S1d. Back-transformed data are summarized in Figure 5B. Together, these results show that MSN mice display heightened activity when in long photoperiods, a finding not seen in the outbred control strain whose in-cage activity appears to be a stable baseline.

To better characterize how MSN mice display heightened activity in long photoperiods, we examined the relative amount of activity occurring in the light period versus the dark period. The dark period results mostly mirrored the 24-hour activity results. found a highly significant strain effect (*F*_1, 41_ = 95.76, *p* = 2.8×10^−12^, η_p_^2^ = 0.700), a highly significant photoperiod effect (*F*_2, 41_ = 33.39, *p* = 2.5×10^−9^, η_p_^2^ = 0.620), and no significant interaction effect (*F*_2, 41_ = 1.59, *p* = 0.22, η_p_^2^ = 0.001) in a two-way ANOVA on inverse square root transformed in-cage velocity for the dark period. Pairwise tests are summarized in Table S1e, and back-transformed data are summarized in Figure 5C. The light period results strongly contrast with the full 24-hour in-cage activity data. Here, there was a highly significant strain effect (*F*_1, 41_ = 19.85, *p* = 6.3×10^−5^, η_p_^2^ = 0.326), a highly significant photoperiod effect (*F*_2, 41_ = 25.55, *p* = 6.2×10^−8^, η_p_^2^ = 0.555), and a significant interaction effect (*F*_2, 41_ = 5.19, *p* = 0.0098, η_p_^2^ = 0.202) in a two-way ANOVA on square root transformed in-cage velocity. Pairwise tests are summarized in Table S1f, and back-transformed data are summarized in Figure 5D.

Altogether, these results suggest that while photoperiod alters the ratio of light period to dark period in-cage activity in ICR mice, that strain's 24-hour in-cage activity remains constant in all photoperiods. In MSN mice, the light to dark in-cage activity ratio is similarly altered, but 24-hour in-cage activity significantly increases in the long photoperiod. As an increase not seen in the control strain, this long photoperiod augmentation of locomotor hyperactivity suggests that MSN have a mania with a comorbid seasonal component. Because mice are nocturnal animals, higher in-cage activity under a shorter dark period is a curious and seemingly paradoxical finding.

## Discussion

### Females

The presentation of the MSN phenotype is sexually dimorphic. Both MSN sexes showed in-cage locomotor hyperactivity relative to outbred control mice, but MSN females displayed significantly higher hyperactivity than their male cohorts. Further, some MSN females displayed total in-cage activity in excess of 1 km, and one female displayed total in-cage activity of greater than 6 km. The origin of this enhanced hyperactivity occurring only in females is unclear. In a previous study on in-cage activity in ICR mice, outbred female mice displayed higher baseline in-cage activity than males [37]. Median female to male activity ratios do not differ significantly between the MSN and ICR strains as studied here (Monte Carlo permutation test, *p* = 0.88, B = 1000), so it appears this normal female-to-male ratio persists in MSN mice. However, the distribution of female MSN mice appears to skew toward the high end much more than the female ICR mice we studied. This sex difference may need more characterization in the future.

In humans, though BPDs show equal prevalence in males and females [38], the outcomes are sexually dimorphic. Relative to men, women with BPDs display later disorder onset [39], tend to cycle more rapidly [40], experience a different subset of comorbid psychiatric disorders [41], and are more prone to mixed manic episodes [42]. Additionally, female reproductive state is associated with disorder presentation [43]. Equal prevalence but differential presentation between the sexes implies that though the genetic basis of BPDs remains constant between the sexes, these heritable underpinnings interact with female physiology differently than they do with male physiology to cause a sexually dimorphic phenotype. Dimorphism in both humans and MSN mice may enhance the face validity of the MSN strain as a mania model, though a more complete phenotyping of female MSN mice will be necessary to examine this hypothesis. Helping characterize the nature of sexual dimorphism in mania presentation may prove an important role for MSN mice.

### Development

From the earliest time point we can reliably record in-cage activity, MSN mice display an observable hyperactive phenotype. The phenotype appears to be stable, affecting mice equally at the full range of dates tested in this study from 4 weeks old to 13 weeks old. In humans, BPDs are often diagnosed in late adolescence to early adulthood [44], though this age of onset is highly-variable [45] and earlier age of disorder onset is a predictor of the severity of BPDs [46]. There has been a recent trend toward the controversial diagnosis of juvenile BPDs [47], [48]. Still, the current consensus on the onset of BPDs appears at odds with our MSN results, a possible caveat to the face validity of the MSN strain.

A contemporary evidence-based theory on the staging of BPDs posits that affective disruptions are rarely observed until early adolescence, but non-affective disruptions predictive of BPDs including hyperactivity, sleep disruptions, and anxiety are observable at very young ages [49]. Additionally, since human BPDs are highly heritable [2], we would argue that these disorders exist latent in humans even at early stages of development. Thus, it is possible that the high in-cage activity we see in even young MSN mice is consistent with pre-bipolar hyperactivity and sleep disruption in humans. In these experiments, we measured locomotion, and spontaneous locomotor activity may not be synonymous with affect. Thus, correlating hyperactivity in emerging human BPDs and in-cage activity in young MSN mice may still be of interest.

### A Unipolar Mania Model

The current evidence suggests that MSN mice do not display a true bipolar phenotype. Instead, they appear stably manic as measured by in-cage locomotor activity. If bipolarism exists in MSN mice, it is difficult to detect. This result is consistent with a previous study on bipolarism in a transgenic mouse line in which only one strong depressed phase was observed in a single mouse as assayed by wheel running [50]. MSN mice may similarly display behavioral bipolarism, but if they do, it is not on any timescale we can reasonably observe.

### Altered Diurnal Activity Profile

BPDs have a high comorbiditiy with altered diurnal preference in humans [51], [52]. Further, total sleep deprivation has antidepressant effects [53], [54], sometimes even throwing patients with BPDs from depression into a manic state [55]. Social rhythm therapy, a new, successful, and non-pharmacological treatment for BPDs, is primarily a chronobiological intervention, implying a strong diurnal constituent to these disorders [56]–[58].

These chronobiological alterations in patients with BPDs may reflect an altered diurnal entrainment mechanism in the brain that is only partially-characterized [59]. There is an association between molecular clock genes and mood disorders in humans [60]–[62], and alterations of molecular clock genes have been used to model BPDs in mice [25]. However, as many molecular clock genes are orphaned receptors [63], the mechanistic nature of altered diurnal preference in BPDs remains unresolved. Nonetheless, there is strong evidence that chronobiology and BPDs are deeply intertwined.

MSN mice display a diurnal activity pattern marked by earlier rising than outbred controls, very high in-cage activity early in the dark period, and a precipitous drop in in-cage activity midway through the dark period. This diurnal activity pattern, an advanced angle of photoentraiment, is potentially analogous to morningness, a tendency to rise earlier and sleep earlier, in humans [64], though the precise physiological correlation to humans is unknown. Because mice are nocturnal and humans are diurnal, precisely determining the correspondence between murine and human diurnal activity patterns is difficult. Regardless, the advantage of having a mania model with a comorbid altered diurnal activity pattern is readily apparent. Chronobiological interventions can be tested to see how altering diurnal patterns might affect mania. The MSN diurnal activity pattern may provide additional predictive validity for these animals in the future, a move toward translation that could represent a fruitful new direction for this strain.

### Photoperiod-Dependent Elevated Hyperactivity and Seasonality

Seasonal affective disorder (SAD) and BPDs have a high comorbidity [65]. Seasonal disruptions in sleep are associated with BPDs [65]. Seasonal decreases in performance on neuropsychological tasks are unusually high in both patients with BPDs and their psychiatrically healthy relatives [66], implying a strongly genetic component to this comorbid seasonality. Additionally, BPDs occur with higher frequency in people born in the winter and in the early spring, though the causality and relevance of this winter-spring birth excess of patients with BPDs are heavily disputed [67].

SAD has few well-validated animal models, and most working models show depression in short days [68]. MSN mice are different, displaying long-day increases of in-cage activity consistent with a seasonally dependent elevation in hyperactivity, the opposite effect seen in other seasonally variable animals. Further, this elevation in activity is not seen in the outbred strain from which MSN mice were derived. Mice are nocturnal, and nocturnal animals moving more in a shortened dark period appears contradictory. This paradoxical long photoperiod increased hyperactivity necessitates some underlying physiological correlate, though we can only speculate about the identity of this component at present. A seasonal constituent unique to the MSN strain could be the result of selection for altered melatonin or kisspeptin systems, which have effects on the GnRH axis and lead to seasonal breeding patterns in other species [69]–[71]. This represents an ethologically based, testable, and parsimonious hypothesis for the physiological basis of the seasonal-like alterations of in-cage activity seen in MSN mice.

### Conclusions and Future Directions

MSN mice are a complex model for the manic pole of BPDs displaying multiple aspects of the human disorders it replicates. Our previous research shows that these mice display a rich suite of behavioral and neural gene expression correlates consistent with a face- and construct-valid mania model. We have also demonstrated that MSN mice display probable genomic perturbations at loci homologous to human loci linked to BPDs, schizophrenia, ADHD, and related disorders. Here, we have added to the corpus of dispositional traits consistent with human BPDs seen in MSN mice. They display sexually dimorphic hyperactivity, an altered chronotype, and photoperiod-dependent increases in hyperactivity. However, unlike humans, the MSN phenotype appears stable during development, and they do not display bipolarism.

The utility of the MSN mouse strain will come from its use as a tool for the translation of basic psychogenetics to human health interventions. Like human BPDs, the MSN phenotype is a complicated one. Understanding the physiological and genetic underpinnings of BPDs is as essential as it is difficult, and we hope that the MSN mouse strain aids in this genetic study. We are currently investigating some of the possible genomic perturbations in these mice seen in the neurogenetic paper we published recently [34]. A partial sequence of the MSN genome is the likeliest path toward novel insights into this phenotype. We are interested in correlating these genotypic differences to human disorders.

## Materials and Methods

### Ethics Statement

Animal use was carried out in accordance with the recommendations in the Guide for the Care and Use of Laboratory Animals of the National Institutes of Health. All protocols were approved by the IACUC for the University of Wisconsin–Madison College of Letters and Sciences (protocol #L00405-0-05-09), and all reasonable efforts were made to minimize animal suffering.

### Animals

The Madison (MSN) mouse strain is a mostly inbred strain derived over a period of approximately 15 years from the outbred hsd:ICR (ICR) strain (Harlan Laboratories, Madison, WI, USA). We estimate the current inbreeding coefficient of the MSN strain at approximately 0.90–0.95. Full details of the inbreeding are described previously [34], [35]. As the genetic background for the MSN strain, the ICR strain is a natural control. We keep breeding colonies of each strain in our laboratory to eliminate as many environmental differences as possible, though we breed new males ordered from Harlan into our ICR colony to prevent this population from experiencing genetic drift. The MSN mouse strain has been submitted to the NIH Mutant Mouse Regional Resource Centers as stock number 036809-MU.

### Behavioral Apparatus

We created a custom experimental apparatus for the behavioral experiments contained in this paper. The apparatus was a 4×4 matrix of clear acrylic home cages in which mice were individually housed modeled after the apparatus used in Zombeck et al. [37]. Cages were manufactured from clear 3/8” acrylic by the shop in the University of Wisconsin–Madison's Department of Zoology. They measured 30.5 cm by 17.7 cm and had steel mesh inserts in the end walls to enhance ventilation. The cages were placed on a black tabletop and purpose-built steel camera rig was placed over the table. Four low-light sensitive security cameras (Panasonic WV-CP284) were placed overhead to capture video.

Since the video ethometry suite we use needs high contrast between the animal and its background in order to analyze position, we used a 1∶1 mixture of the bedding materials Cellu-Dri Soft and PAPERCHIP and added the bedding/enrichment material EnviroDri (Shepherd Specialty Papers, Kalamazoo, MI, USA). These materials are dark grey or brown and are easily distinguished by our software from a white mouse under even the lowest light. To keep glare from disrupting the analysis software, cages were lit from below the table. Compact fluorescent red lights were kept on 24 h a day so that we could collect video during the dark period. Compact fluorescent white lights were put on timers appropriate to the photoperiod chosen for the specific group of mice under study, generally 12 h light and 12 h dark unless otherwise noted. This behavioral apparatus was inspired by the one used by Zombeck et al. [37].

### Software and Statistics

We used TopScan v. 2.00 (CleverSys, Reston, VA, USA) for data analysis as described previously [34], [35] with some important modifications. Instead of using the software's online video analysis capabilities as we did previously, we collected 48 half-hour mpeg files per 24 hours and analyzed them offline from a separate computer networked to the computer collecting the data. Our new behavioral apparatus allowed for us to measure activity continuously for all 24 hours each day instead of 5.5 hours during the light period and 9.5 hours during the dark period as we were doing previously. Additionally, by collecting videos in half-hour increments, we could analyze each half-hour separately and compare behavior at different time points throughout the day. This allowed both continuous data collection and high-resolution analysis of diurnal activity profile.

All statistical analyses were performed using R v. 3.0.1 (x86_64) in OS X v. 10.8.4. Remedial transformations for datasets were chosen using the Box-Cox power transformation method implemented in the R package MASS v. 7.3–23. Type II ANOVAs and effect sizes were calculated using the R package heplots v. 1.0–5. Plots were generated using the R package lattice v. 0.20–13. Welch's two-sample t-tests were generated using the t.test() function in R. All *p*-values are from two-sided tests, and FDR adjustment was done using the p.adjust() function in R.

Upon examination of all 24-hour in-cage activity data contained in this paper, we determined that they were problematically non-normal and heteroscedactic. We found that an inverse square root transformation adequately remediated this problem for 24-hour in-cage activity data. All inferential statistics are performed on data thus transformed unless otherwise noted.

Because any statistical correction is conservative, when presenting pairwise tests, we chose a cutoff for our Tukey HSD *p*-values of 0.10. We note that this is a deviation from the customary *p*-value significance cutoff of 0.05. We checked the nominal significance of each of these tests using two-tailed t-tests, and each was nominally significant at *p*<0.05. We believe that because these data are exploratory, type I errors are more acceptable than type II errors.

### Experiment 1: Female Observations

We were concerned primarily with presence or absence of a phenotype in females. Because of estrous cycling, we believed we would see greater variance in females, so we doubled the replicates we generally used for testing the males' in-cage activity, looking at 16 females from each strain from a single generation. Shortly after behavioral testing, we determined estrous state in females by vaginal lavage. We also looked for sexual dimorphism, comparing these females to equal numbers of age-matched males from each strain.

We used animals aged between postnatal weeks 12 and 13 for this experiment. When we previously looked at 2 days' data for the simple presence or absence of the MSN phenotype, we found the same results for the first day as the second. This is evidence that habituation is unnecessary to observe the effect, so we measured in-cage for the 24 hours directly after putting the animals in the experimental apparatus. All animals used in this experiment were group housed prior to behavioral data collection in a 12 h photoperiod and had ad libitum access to water and food.

### Experiment 2: Developmental Observations

To assess whether the MSN phenotype is present as soon as in-cage activity is easily observable, we designed an experiment that looked at in-cage activity in juvenile male mice shortly after weaning until they reached adulthood. These mice were group housed at 12 h photoperiods and had ad libitum access to food and water. We took 24 hours of behavioral data without habituation each week between postnatal weeks 4 and 7 from 8 MSN mice and 8 ICR mice from the same groups over the course of four weeks. Since the mice were group housed, it was not practical to keep track of individuals. Consequently, we do not have repeated measures for these animals.

### Experiment 3: Month Long Observations

8 MSN and 8 ICR males from the same generation were group housed from weaning to postnatal week 8. The animals were raised under 12 h photoperiods and had ad libitum access to water and food. At 8 weeks, the mice were individually housed in experimental cages. Since a primary purpose for this experiment was to explore bipolarism, we wanted to get stable data for in-cage activity with as few outside influences as possible. Consequently, animals were allowed to habituate for 24 hours prior to collection of in-cage activity data. We collected behavioral data for 28 days in total. We cleaned cages during the light period once every 7 days and excluded the data from that light period and the subsequent dark period from our analysis.

We experienced occasional difficulties during this experiment. In one case (ICR 13), the bedding materials were distributed in an unusual manner, causing a failure in tracking for most of one light period and one dark period. We excluded the affected light and dark periods for this animal. Additionally, the computer collecting the video experienced a blue screen event and crashed for 27 hours at days 21 and 22 of the experiment. We have no data for any animal during this time period.

When looking at probability density for 24-hour activity, we excluded all days for which we did not have a dark period and subsequent light period from our density plots. All observations in these plots contain a full 24 hours of in-cage activity from lights off to lights off. When looking at the diurnal activity pattern, we first found the mean in-cage activity expressed as a velocity for each animal at each time point, averaging all available data from each time point. We used these averages to find group means for both MSN and ICR. These data are double plotted with standard errors of the mean as ribbons around the lines.

### Experiment 4: Photoperiod Observations

To assay effects of photoperiod, we took mice directly from weaning and group housed them in rooms with 3 different photoperiods. A group of 16 male mice, 8 MSN and 8 ICR each, was housed in each the following photoperiods: 6 h of light (6∶18 L∶D), 12 h of light (12∶12 L∶D), and 18 h of light (18∶6 L∶D). Mice were group housed in these photoperiods until they were aged 12–13 weeks. Before the experiment, one mouse from the MSN 6 h photoperiod group died, so we only ended up testing 7 MSN mice and 8 ICR mice from that photoperiod. We collected behavioral data in the same photoperiod in which the mice were raised for 24 hours after an hour of habituation.

## Supporting Information

Table S1 Supplementary tables containing statistical test results.
(PDF)
