# Supplementary material for: Sexually Dimorphic, Developmental, and Chronobiological Behavioral Profiles of a Mouse Mania Model
Source: PLoS One. 2013 Aug 13;8(8):e72125. doi: 10.1371/journal.pone.0072125 (PMC3742520; doi:10.1371/journal.pone.0072125)
Supplement: Table S1 — Supplementary tables containing statistical test results. (PDF) [file pone.0072125.s001.pdf]

Supplementary Tables S1: Pairwise Post-Hoc Tests

S1a: Females Pairwise Post-Hoc Tests with 95% CI (Tukey HSD on inverse square root transformed data)

Comparison	Difference	Lower	Upper	*p*-value
MSN:F-ICR:F	-0.015598	-0.0214177	-0.009779	1.1×10^{-8}
MSN:M-ICR:M	-0.014959	-0.0207783	-0.00914	3.4×10^{-8}
MSN:M-MSN:F	0.005587	-0.0002326	0.011406	0.0643
ICR:M-ICR:F	0.004947	-0.0008719	0.010766	0.1225

S1b: Development Pairwise Post-Hoc Tests with 95% CI (Tukey HSD on inverse square root transformed data)

Comparison	Difference	Lower	Upper	*p*-value
MSN:WK7-ICR:WK7	-0.0178885	-0.026497	-0.00928	5.4×10^{-7}
MSN:WK6-ICR:WK6	-0.0175835	-0.026192	-0.008975	8.2×10^{-7}
MSN:WK5-ICR:WK5	-0.0114328	-0.0200413	-0.0028243	0.0025
ICR:WK7-ICR:WK4	0.0088372	0.0002287	0.0174458	0.0402
MSN:WK4-ICR:WK4	-0.0083446	-0.0169531	0.0002639	0.0638
ICR:WK7-ICR:WK5	0.0056289	-0.0029796	0.0142374	0.4535
ICR:WK6-ICR:WK4	0.0052136	-0.0033949	0.0138222	0.5523
MSN:WK6-MSN:WK5	-0.0041454	-0.0127539	0.0044631	0.7956
MSN:WK6-MSN:WK4	-0.0040253	-0.0126338	0.0045832	0.8186
ICR:WK7-ICR:WK6	0.0036236	-0.0049849	0.0122321	0.8853
MSN:WK7-MSN:WK6	0.0033186	-0.0052899	0.0119272	0.9245
ICR:WK5-ICR:WK4	0.0032083	-0.0054002	0.0118168	0.9362
ICR:WK6-ICR:WK5	0.0020053	-0.0066032	0.0106138	0.9956
MSN:WK7-MSN:WK5	-0.0008267	-0.0094353	0.0077818	1
MSN:WK7-MSN:WK4	-0.0007066	-0.0093151	0.0079019	1
MSN:WK5-MSN:WK4	0.0001201	-0.0084884	0.0087286	1

S1c: Diurnal Activity Profile Formal Tests (FDR-adjusted Welch's t-tests)

Comparison	df	t	*p*-value	FDR *p*-value
MSN:0000-ICR:0000	10.8625	3.7046	0.0035	0.0156
MSN:0030-ICR:0030	9.4326	2.2739	0.0478	0.0882
MSN:0100-ICR:0100	10.2195	1.8069	0.1003	0.1553
MSN:0130-ICR:0130	11.3864	1.1362	0.2792	0.3351
MSN:0200-ICR:0200	11.1498	0.6328	0.5397	0.6168
MSN:0230-ICR:0230	9.1335	0.2340	0.8202	0.8558
MSN:0300-ICR:0300	9.4088	0.1800	0.8610	0.8723

Comparison	df	t	*p*-value	FDR *p*-value
MSN:0330-ICR:0330	9.4905	-0.5035	0.6261	0.6830
MSN:0400-ICR:0400	9.0252	-0.6976	0.5030	0.5889
MSN:0430-ICR:0430	9.5648	-1.7585	0.1106	0.1608
MSN:0500-ICR:0500	8.5383	-1.8700	0.0961	0.1537
MSN:0530-ICR:0530	7.5738	-2.4706	0.0403	0.0773
MSN:0600-ICR:0600	7.9807	-1.9193	0.0913	0.1511
MSN:0630-ICR:0630	8.2112	-1.4998	0.1711	0.2161
MSN:0700-ICR:0700	13.2310	0.6078	0.5536	0.6180
MSN:0730-ICR:0730	11.7164	1.7360	0.1087	0.1608
MSN:0800-ICR:0800	12.3558	1.6154	0.1314	0.1705
MSN:0830-ICR:0830	13.0423	-0.1639	0.8723	0.8723
MSN:0900-ICR:0900	9.7499	0.4394	0.6700	0.7146
MSN:0930-ICR:0930	12.0728	1.3673	0.1964	0.2418
MSN:1000-ICR:1000	13.9705	2.6372	0.0195	0.0461
MSN:1030-ICR:1030	9.8754	1.7086	0.1187	0.1628
MSN:1100-ICR:1100	13.0698	4.0920	0.0013	0.0132
MSN:1130-ICR:1130	13.8564	2.4720	0.0270	0.0564
MSN:1200-ICR:1200	11.6686	1.7065	0.1144	0.1615
MSN:1230-ICR:1230	12.6643	2.0376	0.0630	0.1121
MSN:1300-ICR:1300	13.3286	2.6372	0.0202	0.0461
MSN:1330-ICR:1330	10.5290	2.8485	0.0165	0.0416
MSN:1400-ICR:1400	10.4584	3.3364	0.0071	0.0243
MSN:1430-ICR:1430	11.2801	4.0498	0.0018	0.0135
MSN:1500-ICR:1500	10.6350	3.5080	0.0052	0.0190
MSN:1530-ICR:1530	13.4362	4.3053	0.0008	0.0132
MSN:1600-ICR:1600	12.3255	3.6420	0.0032	0.0156
MSN:1630-ICR:1630	10.0748	3.7773	0.0036	0.0156
MSN:1700-ICR:1700	9.3474	1.6672	0.1286	0.1705
MSN:1730-ICR:1730	10.7564	2.3647	0.0380	0.0760
MSN:1800-ICR:1800	12.8335	1.9651	0.0714	0.1224
MSN:1830-ICR:1830	13.9846	2.8871	0.0120	0.0359
MSN:1900-ICR:1900	12.3519	3.8377	0.0022	0.0135
MSN:1930-ICR:1930	12.2466	4.4840	0.0007	0.0132
MSN:2000-ICR:2000	10.6868	4.0201	0.0021	0.0135
MSN:2030-ICR:2030	12.0068	4.1451	0.0014	0.0132
MSN:2100-ICR:2100	13.7290	3.9947	0.0014	0.0132
MSN:2130-ICR:2130	13.2789	2.7643	0.0158	0.0416
MSN:2200-ICR:2200	11.3910	2.6283	0.0229	0.0499
MSN:2230-ICR:2230	12.2839	2.8369	0.0147	0.0415
MSN:2300-ICR:2300	11.5292	3.0689	0.0102	0.0325
MSN:2330-ICR:2330	11.2343	3.4821	0.0050	0.0190

S1d: 24-hour Photoperiod Pairwise Post-Hoc Tests with 95% CI (Tukey HSD on inverse square root transformed data)

**Comparison**	**Difference**	**Lower**	**Upper**	***p*-value**
MSN:18H-ICR:18H	-0.019533	-0.02737	-0.0116969	5.6×10^{-8}
MSN:6H-ICR:6H	-0.013802	-0.021914	-0.0056904	1.2×10^{-4}
MSN:12H-ICR:12H	-0.013759	-0.021595	-0.0059221	7.1×10^{-5}
MSN:18H-MSN:12H	-0.012635	-0.020471	-0.0047985	2.7×10^{-4}
MSN:18H-MSN:6H	-0.007329	-0.01544	0.0007828	0.097
ICR:18H-ICR:12H	-0.00686	-0.014697	0.0009764	0.1162
ICR:12H-ICR:6H	0.005263	-0.002574	0.0130994	0.356
MSN:12H-MSN:6H	0.005306	-0.002805	0.0134178	0.3849
ICR:18H-ICR:6H	-0.001597	-0.009434	0.0062392	0.9898

S1e: Dark Photoperiod Pairwise Post-Hoc Tests with 95% CI (Tukey HSD on inverse square root transformed data)

**Comparison**	**Difference**	**Lower**	**Upper**	***p*-value**
MSN:18H-ICR:18H	-0.081822	-0.11684	-0.0468	2.5×10^{-7}
MSN:18H-MSN:12H	-0.077636	-0.11265	-0.04262	8.1×10^{-7}
MSN:6H-ICR:6H	-0.066025	-0.10227	-0.02978	3.8×10^{-5}
MSN:18H-MSN:6H	-0.061065	-0.09731	-0.02482	1.4×10^{-4}
MSN:12H-ICR:12H	-0.052935	-0.08795	-0.01792	7.0×10^{-4}
ICR:18H-ICR:12H	-0.048748	-0.08377	-0.01373	0.0021
ICR:18H-ICR:6H	-0.045267	-0.08029	-0.01025	0.0049
MSN:12H-MSN:6H	0.016571	-0.01968	0.05282	0.7464
ICR:12H-ICR:6H	0.003481	-0.03154	0.0385	0.9997

S1f: Light Period Pairwise Post-Hoc Tests with 95% CI (Tukey HSD on square root transformed data)

**Comparison**	**Difference**	**Lower**	**Upper**	***p*-value**
MSN:18H-MSN:6H	2.50916	1.47134	3.547	1.2×10^{-7}
MSN:18H-ICR:18H	1.60238	0.59974	2.605	3.1×10^{-4}
MSN:18H-MSN:12H	1.39357	0.39093	2.3962	0.0021
MSN:12H-MSN:6H	1.1156	0.07777	2.1534	0.0287
ICR:18H-ICR:6H	0.95474	-0.04789	1.9574	0.0699
MSN:12H-ICR:12H	0.9125	-0.09014	1.9151	0.0929
ICR:18H-ICR:12H	0.70369	-0.29894	1.7063	0.3087
ICR:12H-ICR:6H	0.25105	-0.75158	1.2537	0.9744
MSN:6H-ICR:6H	0.04795	-0.98987	1.0858	1
